# A systematic review of Tuina for cervical hypertension: A protocol for systematic review and meta-analysis

**DOI:** 10.1097/MD.0000000000030699

**Published:** 2022-10-07

**Authors:** Hongyi Guan, Haiyu Zhu, Jiaxin Gao, Tingwei Ding, Qin Wu, Yunpeng Bi, Yufeng Wang, Xingquan Wu, Bailin Song

**Affiliations:** a Department of Acupuncture and Tuina, Changchun University of Chinese Medicine, Changchun, China; b Department of Chinese and Western Integrative Medicine, Liaoning University of Chinese Medicine, Liaoning, China; c Department of Tuina, Changchun University of Chinese Medicine, Changchun, China; d Traditional Chinese Medicine, Changchun University of Chinese Medicine, Changchun, China.

**Keywords:** cervical hypertension, meta-analysis, protocol, systematic review, Tuina

## Abstract

Cervical hypertension is a secondary form of hypertension with a high incidence rate. As the main etiology of cervical hypertension is related to cervical spondylosis, commonly used antihypertensive drugs have poor efficacy in the treatment of cervical spondylosis, and improving the symptoms of cervical spondylosis can effectively reduce blood pressure. Massage can effectively improve the symptoms of patients with cervical hypertension, but there has been no systematic review of massage treatment for cervical hypertension. This study aimed to evaluate the efficacy and safety of massage in patients with cervical hypertension.

**Methods:**

Before February 10, 2022, a systematic literature search was conducted using the following databases: Embase, SinoMed (previously called the Chinese Biomedical Database), China Science and Technology Journal Database for Chinese Technical Periodicals, Chinese National Knowledge Infrastructure, and Wanfang Data. Review Manager software (version 5.3) will be used for statistical analysis. Quality and risk assessments of the included studies were performed, and the outcome indicators of the trials were observed.

**Results:**

This meta-analysis further confirmed the beneficial effects of massage in patients with cervical hypertension.

**Conclusion:**

This study investigated the efficacy and safety of massage therapy in patients with cervical hypertension, providing clinicians and patients with additional options for the treatment of this disease.

## 1. Introduction

Hypertension is an important medical problem worldwide,^[1,2]^ affecting the health of 1 billion people worldwide, and is a key factor leading to diseases of the heart, kidneys, and other important organs.^[3]^ In China, according to screening results from 2014 to 2017, the prevalence of hypertension in Chinese adults was as high as 44.7%.^[4]^ Hypertension can lead to various diseases and is associated with a high mortality rate.^[5]^ Patients with hypertension require long-term continuous medication if they take medication, but these drugs often lead to hyperkalemia, sexual dysfunction, decreased renal function, and other serious adverse reactions, which greatly affect the quality of life of patients.^[6]^

An increasing number of studies have found a close relationship between cervical spondylosis and hypertension, attracting increasing attention.^[7–9]^ Since the 1970s, there have been reports of abnormal cervical blood pressure, the main cause of which is an increase in blood pressure caused by cervical spondylosis.^[10]^ Cervical hypertension is a secondary hypertension with a high incidence, and its pathogenesis is unclear. It is generally believed that the pathogenesis of cervical spine strain degeneration, other mechanical injuries, and inflammatory substances leads to increased blood pressure.^[11–13]^

In the treatment of patients with cervical hypertension, the effect of antihypertensive drugs was not obvious.^[14]^ The changes in blood pressure of patients are often synchronized with the changes in symptoms of cervical spondylosis; therefore, treatment for the etiology of cervical spondylosis can help patients effectively control their blood pressure.^[15,16]^ Tuina is an external treatment of traditional Chinese medicine and has the effect of relaxing tendons and dreading collaterals, activating qi and blood circulation, correcting bone joint dislocation, restoring the normal sequence of the cervical vertebra, restoring the normal function of the sympathetic nerve vertebral artery, and regulating blood pressure.^[17,18]^

However, there are no systematic evaluation reports on the efficacy and safety of massage in the treatment of cervical hypertension. However, there are no systematic evaluation reports on the efficacy and safety of massage in the treatment of cervical hypertension. However, there are no systematic evaluation reports on the efficacy and safety of massage in the treatment of cervical hypertension.

## 2. Methods

The system evaluation registration number (CRD42022295810) was registered in the PROSPERO International System Evaluation Prospective Registry. All steps in this systematic review were performed according to the Cochrane manual (version 5.2.0).

### 2.1. Eligibility criteria

#### 2.1.1. Type of study.

A systematic review of all randomized controlled trials was conducted to evaluate the efficacy and safety of massage therapy for hypertension with cervical spondylosis. This study included both published and unpublished randomized controlled trials, regardless of blindness and language restrictions.

#### 2.1.2. Types of participants.

Participants had been diagnosed with hypertension complicated by cervical spondylosis, regardless of the disease course and severity, and met the following criteria.

1. Diagnostic criteria of hypertension: systolic blood pressure ≥ 140 mm Hg or diastolic blood pressure ≥ 90 mm Hg;
2. Diagnostic criteria for cervical spondylosis: Diagnosis of cervical spondylosis using computed tomography, magnetic resonance imaging, and other imaging methods.

#### 2.1.3. Types of interventions and comparisons.

The main intervention method in this study was Tuina, which also included massage, manipulation, traditional Chinese massage, and other similar methods. Trials that combined massage and other treatments were also included. There was no limit to the time or frequency of massage. Patients in the control group received acupuncture, drugs, placebos, or no treatment.

#### 2.1.4. Outcomes.

The results were effective, including decreased blood pressure, improved cervical symptoms, and an overall improvement (including blood pressure and cervical spondylosis). Systolic and diastolic pressures decreased and negative effects were observed.

### 2.2. Exclusion criteria

The exclusion criteria contain the following items:

1. Essential hypertension.
2. Secondary hypertension caused by craniocerebral injury, kidney, adrenal gland, and goiter.
3. Patients with congenital heart disease or organ dysfunction.
4. Pregnant and lactating women.

### 2.3. Search strategy

Our research group plans to search the Embase, SinoMed (previously called the Chinese Biomedical Database), China Science and Technology Journal Database for Chinese Technical Periodicals, Chinese National Knowledge Infrastructure, and Wanfang Data databases. From the beginning of the study to February 2022, all published randomized controlled trials in Chinese and English were included. The retrieval mode used will be a combination of free words and medical subject headings terms, including “hypertension,” “blood pressure, high,” “blood pressures, high,” “high blood pressure,” “cervical vertebra,” “vertebrae,” “cervical,” “tuina,” “massage,” “Chinese massage,” “Chinese manipulation,” “maasge therapy.” The search strategy used PubMed as an example (Table 1).

**Table 1:** Search strategy for the PubMed database.

Number	Terms
#1	hypertension (Mesh Terms)
#2	blood pressure, high (Title/Abstract)
#3	blood pressures, high (Title/Abstract)
#4	high blood pressure (Title/Abstract)
#5	high blood pressures (Title/Abstract)
#6	OR #1-#5
#7	cervical vertebra (Mesh Terms)
#8	Vertebrae (Title/Abstract)
#9	cervical (Title/Abstract)
#10	OR #7-#9
#11	Tuina (all field)
#12	Chinese Tuina (all field)
#13	Massage (all field)
#14	Massage therapy (all field)
#15	Chinese massage (all field)
#16	Manipulation (all field)
#17	Chinese manipulation therapy (all field)
#18	Chinese manipulation (all field)
#19	OR #11-#18
#20	Randomized controlled trial (all field)
#21	Controlled clinical trial (all field)
#22	Randomly (all field)
#23	Randomized (all field)
#24	Random allocation (all field)
#25	Placebo (all field)
#26	Double-blind method (all field)
#27	single-blind method (all field)
#28	Trials (all field)
#29	OR #20-28
#30	#6 And #10 And #19 And #29

### 2.4. Data collection and analysis

#### 2.4.1. Selection of studies.

**Figure 1.**
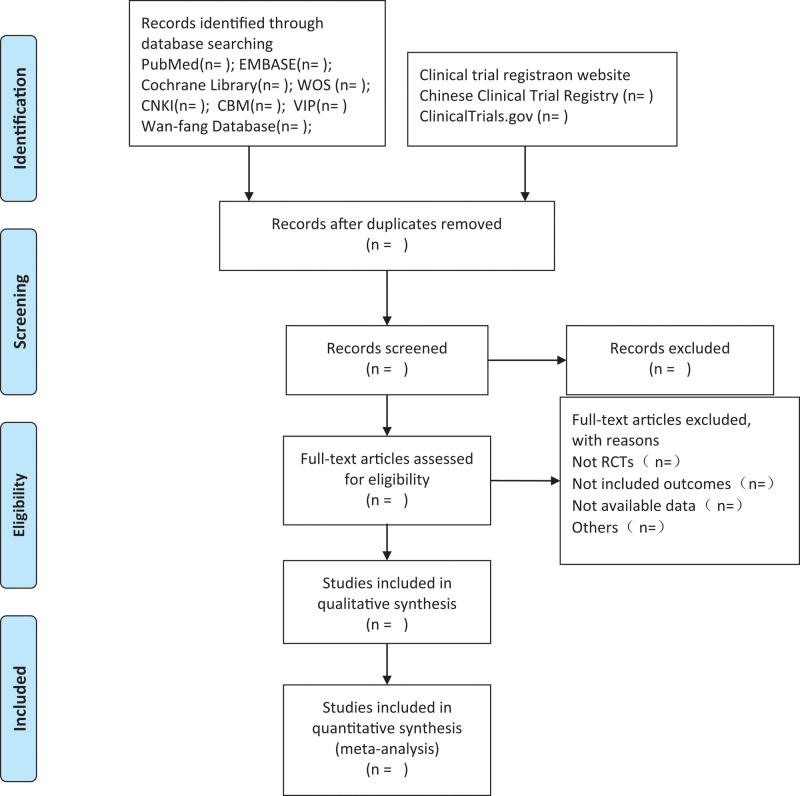
Flow diagram of study selection process.

First, 3 members (GHY, DTW, and ZHY) of the research group will independently screen eligible studies. The selected research was imported into the document management software EndNote X9. Second, preliminary screening was conducted according to the title and summary, and the appropriate research was selected according to the inclusion criteria. Finally, it was decided that if there were differences in inclusion and exclusion, the final conclusion would be drawn through group discussions. This process is shown in the PRISMA follow-up diagram (Fig. 1).

#### 2.4.2. Data extraction and management.

Two authors (GHY and DTW) extracted data from eligible studies. The extracted data included the author’s name, title, country, publication date, research design, age and sex of participants, course of disease, details of control interventions, time, results, follow-up, adverse reactions, and quality of life. The extracted data were entered into the electronic database by 2 authors, and if a difference occurred, it was verified by a third party (GJX).

#### 2.4.3. Dealing with missing data.

In the face of missing data or unclear research, we contacted the relevant authors to obtain missing information before making an exclusion decision. If true information acquisition failed, we excluded it from the analysis.

### 2.5. Risk of bias assessment

The Cochrane^[19]^ Collaboration tool was used to assess the risk of bias in each study, including the following 6 types of bias: random sequence generation, allocation concealment, participant and personal blinding, outcome assessment blinding, incomplete outcome data, selective reporting, and other sources of bias. The quality of the report was divided into 3 levels: low, unclear, and high-risk. Differences were resolved through group discussion.

### 2.6. Statistical analysis

This study will be analyzed using RevMan version 5.3. Relative risk (RR) was used when the results were dichotomous variables with 95% confidence intervals. For continuous variables, we used the standardized mean difference and 95% confidence intervals. The chi-square test and I2 statistic will be used to confirm heterogeneity. The former checks for heterogeneity, whereas the latter reflects the degree of heterogeneity through a specific value. *I*^2^ values of 25%, 50%, and 75% indicated low, medium, and high heterogeneity, respectively. If *I*^2^ was >50%, there was considerable heterogeneity between the studies; therefore, a subgroup analysis was performed to investigate the potential causes.

### 2.7. Sensitivity analysis

In terms of quality analysis, we conducted a sensitivity analysis on the main results to explore the impact of individual research bias on the results.

### 2.8. Subgroup analysis

If heterogeneity was obvious, according to the characteristics of this study, subgroup analysis was performed to explore the sources of heterogeneity from the aspects of age, sex, region, control intervention type, and massage type.

## 3. Discussion

Hypertension is a common and multiple global disease that poses a life-threatening economic burden to human beings. Previous studies have shown that cervical hypertension is the cause of many diseases, and the efficacy of commonly used antihypertensive drugs in the treatment of this disease is unclear.^[20,21]^ Tuina, as a traditional external therapy in traditional Chinese medicine, can effectively reduce the blood pressure of patients by correcting the dislocation of the neck joints, releasing rigid muscles, and promoting blood circulation.^[22,23]^ However, no systematic review has been conducted to date. This study is the first randomized controlled trial to evaluate the efficacy and safety of massage therapy for the treatment of hypertension with cervical spondylosis.

Our research team will objectively and comprehensively evaluate the therapeutic effect of massage therapy on hypertensive patients with cervical spondylosis to reduce blood pressure and improve symptoms of cervical spondylosis. The results of this review will provide chiropractors, cardiologists, and patients with more information on treatment options for cervical hypertension and new directions for future research based on the credibility of existing evidence.

## Author contributions

Hongyi Guan and Haiyu Zhu had the original idea of this work and drafted the protocol. The search strategy was developed by all authors and will be performed by Guan, Gao, Ding, Bi, Wu, et al Song proposed advice for design and revision. Gao and Ding independently collected and extracted eligible studies. Bi and Wu assessed the bias risk and dealt with missing data. All authors who participated in this study critically revised the final version of the manuscript and confirmed the publication of the protocol.

**Conceptualization:** Hongyi Guan, Haiyu Zhu.

**Data curation:** Jiaxin Gao, Tingwei Ding, Yunpeng Bi.

**Formal analysis:** Haiyu Zhu.

**Funding acquisition:** Bailin Song.

**Investigation:** Xingquan Wu, Yufeng Wang.

**Methodology:** Hongyi Guan.

**Supervision:** Bailin Song.

**Validation:** Xingquan Wu, Yufeng Wang.

**Writing – original draft:** Hongyi Guan.

**Writing – review & editing:** Hongyi Guan, Haiyu Zhu, Bailin Song.
